# Artificial Antigen Presenting Cells for Detection and Desensitization of Autoreactive T cells Associated with Type 1 Diabetes

**DOI:** 10.1021/acs.nanolett.2c00819

**Published:** 2022-05-26

**Authors:** Arbel Artzy-Schnirman, Enas Abu-Shah, Rona Chandrawati, Efrat Altman, Norkhairin Yusuf, Shih-Ting Wang, Jose Ramos, Catherine S. Hansel, Maya Haus-Cohen, Rony Dahan, Sefina Arif, Michael L. Dustin, Mark Peakman, Yoram Reiter, Molly M. Stevens

**Affiliations:** † Department of Materials, Department of Bioengineering and Institute for Biomedical Engineering, Imperial College London, Prince Consort Road, London SW7 2AZ, U.K.; ‡ Kennedy Institute of Rheumatology, Nuffield Department of Orthopaedics, Rheumatology and Musculoskeletal Sciences, University of Oxford, Oxford OX3 7FY, U.K.; § Sir William Dunn School of Pathology, University of Oxford, Oxford OX1 3RE, U.K.; ∥ Laboratory of Molecular Immunology, Faculty of Biology and Technion Integrated Cancer Center, Technion-Israel Institute of Technology, Haifa 3200003, Israel; ⊥ Department of Immunobiology, Guy’s, King’s & St Thomas’ School of Medicine, second Floor, New Guy’s House, Guy’s Hospital, London SE1 9RT, U.K.; # Department of Systems Immunology, Weizmann Institute of Science, Rehovot 761001, Israel

**Keywords:** Type 1 diabetes, artificial antigen presenting cells, auto reactive T-cells, Layer-by-layer particles

## Abstract

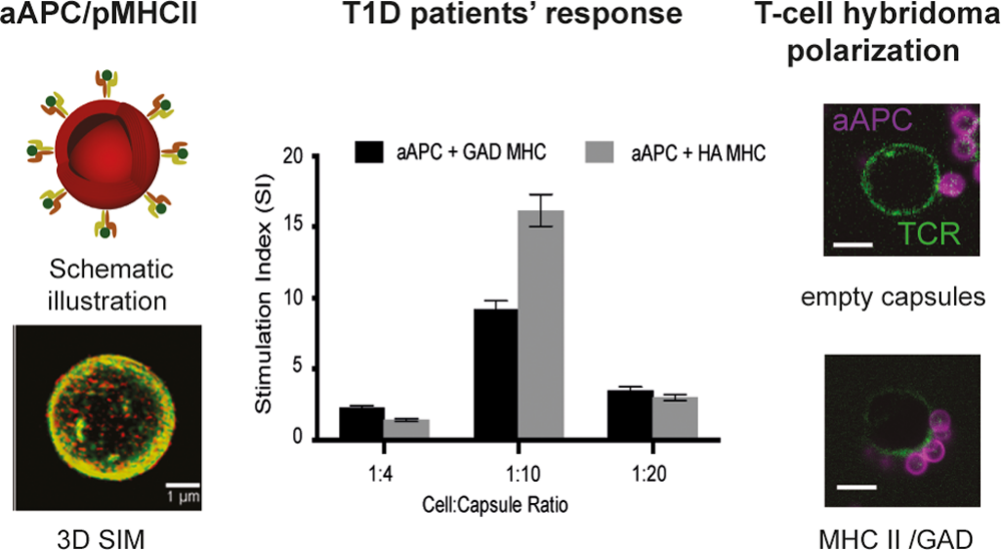

Autoimmune diseases and in particular type 1 diabetes rely heavily on treatments that target the symptoms rather than prevent the underlying disease. One of the barriers to better therapeutic strategies is the inability to detect and efficiently target rare autoreactive T-cell populations that are major drivers of these conditions. Here, we develop a unique artificial antigen-presenting cell (aAPC) system from biocompatible polymer particles that allows specific encapsulation of bioactive ingredients. Using our aAPC, we demonstrate that we are able to detect rare autoreactive CD4 populations in human patients, and using mouse models, we demonstrate that our particles are able to induce desensitization in the autoreactive population. This system provides a promising tool that can be used in the prevention of autoimmunity before disease onset.

## Introduction

Type 1 diabetes (T1D) is one of the major health challenges of the 21st century.^1^ Despite the vast research conducted and the knowledge gained regarding T1D, there is no practical knowledge about prevention and the detailed pathogenesis is not yet fully understood. T1D symptoms appear when the β-cells in the pancreas are so severely damaged that they can no longer produce insulin. Destruction of the β-cells is usually a gradual process, which lasts several years. T1D progress depends both on the genetic background and on environmental conditions.^2^ CD4 T-cells play a central role in the initiation and progression of the disease, as exemplified by the observation that major histocompatibility complex (MHC) II cluster variants are the most associated risk factors with T1D^3^ and other disease risks related to the function of CD4. In T1D autoantibodies targeted against the β-cells appear in the blood up to several years prior to onset of the disease. During this time frame, an early diagnosis of the disease is possible by tracking these antibodies together with well-known genetic markers. So far only the symptoms are being treated, not the disease itself, and currently there is no safe and effective treatment that is approved by the Food and Drug Administration (FDA). One promising approach in the development of therapies for T1D and other autoimmune diseases is to target autoreactive T-cells and to generate specific immune tolerance while keeping the ability to respond to pathogenic antigens.^4^ Novel approaches for immunotherapy include the use of nanoparticles (NPs) coated with peptides and pMHC,^5^ which have been used to target CD8 and regulatory Tr1 cells in mouse models.^6^ The use of pMHC NPs provides support to the use of biosynthetic materials for autoimmune diseases. However, NPs suffer from small size and increased clearance, which may require continuous administration; also they have a small surface area hence a limited density range of antigen and potentially other costimulatory molecules. Also, they do not offer an easy way to encapsulate any soluble factors to be concomitantly introduced (e.g. inhibitory cytokines such as IL10 and TGFbeta). Here we propose to develop artificial antigen presenting cells (aAPCs) based on a layer-by-layer technology as a novel immunotherapeutic approach to manipulate and detect rare autoreactive T-cells within the precious time window before onset of the disease, with the ultimate goal being prevention of the destruction of β-cells. We report the development of unique multifunctional particle carriers presenting MHC II loaded with the GAD_555-567_ peptide, an efficient naturally processed immunodominant T-cell epitope from glutamate decarboxylase in patients with T1D. Our aAPCs have a hollow interior that can be developed to encapsulate biologically active materials to further manipulate the immune responses and target different cell populations. The proposed aAPCs benefit from a high avidity presentation of the peptide-MHC (pMHC) and stiffnesses within the physiological range, which allows optimal desensitization and detection of previously undetectable autoreactive T-cell populations in T1D patients. We also demonstrate that the particles are able to reach the spleen *in vivo* and that they are able to induce desensitization of autoreactive T-cells in a specific manner *ex vivo*. Our targeted applications are (1) early diagnosis in patients with active T1D and (2) tolerance to autoreactive T-cells before β-cells are destroyed.

## Results

**Figure 1:**
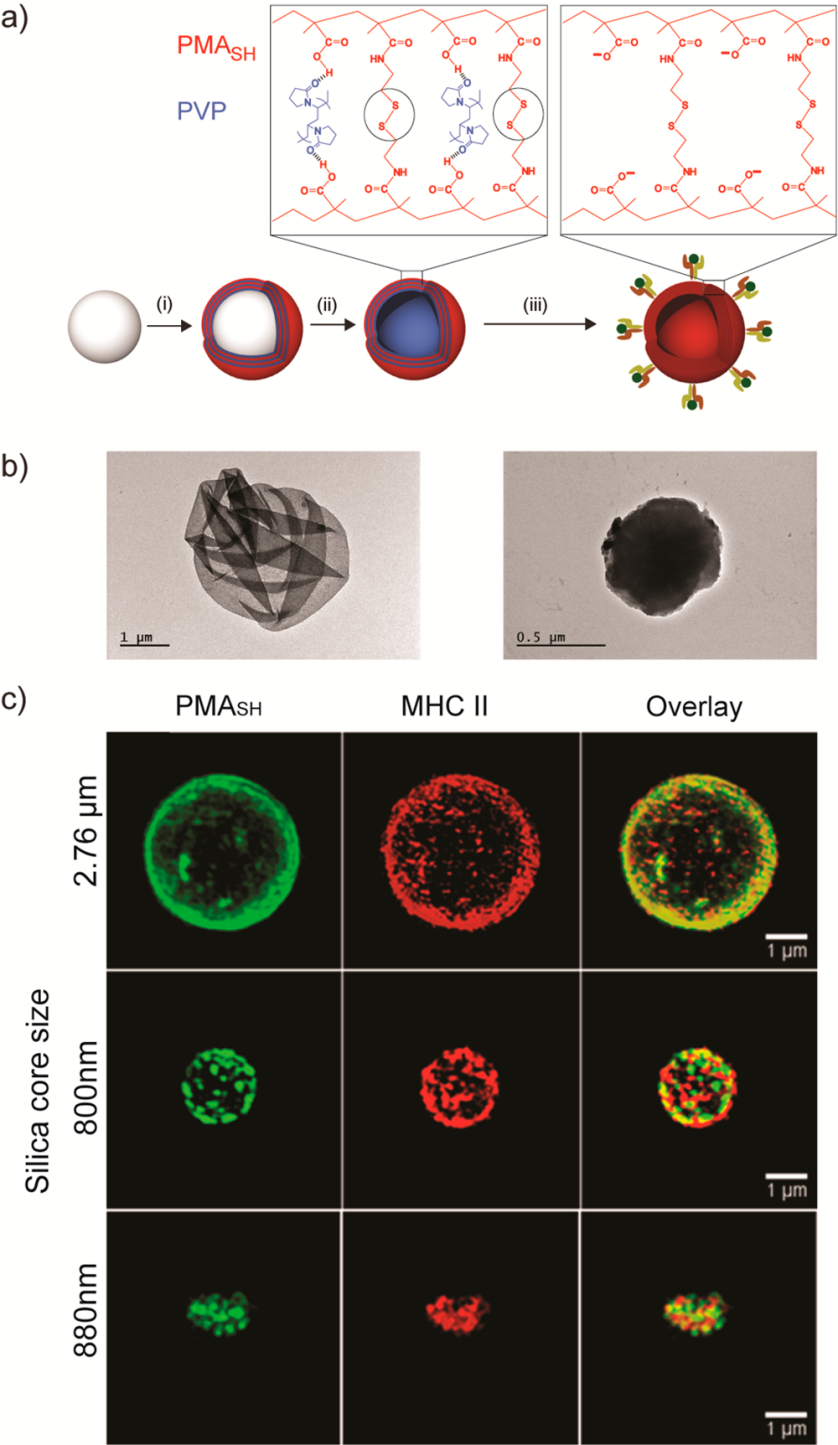
Generation of aAPC using LbL-assembled polymer particles. (a) Schematic illustration of the artificial antigen presenting cell preparation: a sililica core is functionalized; (i) multilayers of PMA_SH_/PVP polymers are assembled on a silica particle template; (ii) the PMA_SH_ layers are then stabilized and cross-linked through disulfide bonds followed by the removal of the silica core, resulting in a hollow polymer capsule; (iii) an additional layer of PMA_SH_ is added to allow the covalent conjugation of MHC II via a disulfide linker to the capsule. (b) TEM images of capsules assembled using 2.76 μm solid-core silica (left) and porous silica (right). (c) 3D structured illumination microscopy (3D-SIM) reconstruction corresponding to particles assembled on 2.76 μm (top) and 800 nm solid-core silica (middle), and on 880 nm porous silica core (bottom). Color code: green, Alexa Fluor 488 labeled PMA_SH_; red, MHC II labeled with PE; yellow, overlay of the MHC II and polymers.

With the aim of abrogating the immune-mediated destruction of β-cells, preserving residual β-cell function in recent-onset T1D and preventing the onset of the disease and islet-specific autoimmunity in at-risk or prediabetes patient populations, we have set out to develop aAPCs based on polymer carrier particles fabricated using layer-by-layer (LbL) techniques^7−9^ with the sequential deposition of interacting polymers onto sacrificial templates. This technique is a facile and low-cost method for forming multilayer polymer films and allows encapsulation of a variety of active (bio)molecules. This technique facilitates control over the size, shape, composition, and permeability of the polymer membrane to allow the controlled release of encapsulated molecules and density of surface molecules presented. Hollow polymer particles made of these polymer pairs have previously been developed to encapsulate DNA,^10,11^ chemotherapeutic drugs,^12−14^ enzymes,^15−17^ and peptide vaccines^18,19^ and used to stimulate T-cells for vaccination *in vitro* and *in vivo*.^20,21^ LbL-assembled particles therefore outperform competing technologies by the simplicity of their assembly, exquisite control over their properties, and demonstrated potential in the area of therapeutic delivery.^12,14,22^ In this study, the polymer building blocks poly(*N*-vinylpyrrolidone) (PVP) and thiol-functionalized poly(methacrylic acid) (PMA_SH_), were chosen with stability, biodegradability, tunable cross-linking density, and surface functionalization in mind (Figure 1a). We used three different silica cores, varying in sizes of 880 ± 89 nm for porous silica and 800 ± 40 and 2760 ± 120 nm solid silica cores and varying porosities, as templates for polymer multilayers, allowing us to explore various antigen loads (see details in Methods in the Supporting Information). The porous silica offers the advantage of creating a capsule with a high surface area due to a higher deposition of the polymer during the layer assembly. It also has the possibility of loading soluble factors and small molecules into the core of the capsule that can later exhibit controlled diffusion and deliver additional signals to the cells. Using fluorescently labeled PMA_SH_, we assessed the increase in polymer deposition as a function of layering rounds (Figure S1). Fluorescence microscopy images show the successful sequential deposition of PVP and fluorescently labeled PMA_SH_ via hydrogen-bonding interactions at pH 4 on porous silica or solid silica cores (Figure S1). Hollow polymer particles were obtained by the dissolution of the silica cores. Upon exposure to physiological conditions (pH 7.4), the hydrogen bonding between PVP and PMA_SH_ diminishes, yielding disulfide-cross-linked PMA particles, as demonstrated by the shift in the ζ potential of the particles (Figure S2). As can be seen from the TEM images, the hollow polymer particles obtained using porous silica templates have a denser appearance (Figure 1b, right) in comparison to those obtained using 2.76 μm solid silica templates (Figure 1b, left), due to the larger amount of polymers deposited onto porous silica cores which have a larger surface area. Similar results were obtained on comparison to the 800 nm solid core (data not shown). Following the preparation of the multilayered polymer particles, an additional layer of PMA_SH_ was added to introduce free surface thiol groups, which can be used to covalently couple proteins. We expressed three different versions of HLA-DR4(*04:01) containing a free cysteine at the C-terminus of the α-chain, which allowed site-specific orientation to the polymer particles: a DR4 complex covalently linked to the T1D associated GAD_555–567_ peptide, a DR4 complex covalently linked to the flu virus hemagglutinin epitope HA_306-318_ as a control, and an “empty” DR4 complex that did not have any covalent peptide (Figure S3). The peptides were covalently bound to the β-chain (Figure S3). Using 3D structured illumination microscopy (3D-SIM), we visualized the presentation of functional pMHC, using a conformation-sensitive antibody (Figure 1c and Figure S4, middle panel), onto fluorescent polymer particles of various sizes (Figure 1c and Figure S4, left panel).

**Figure 2:**
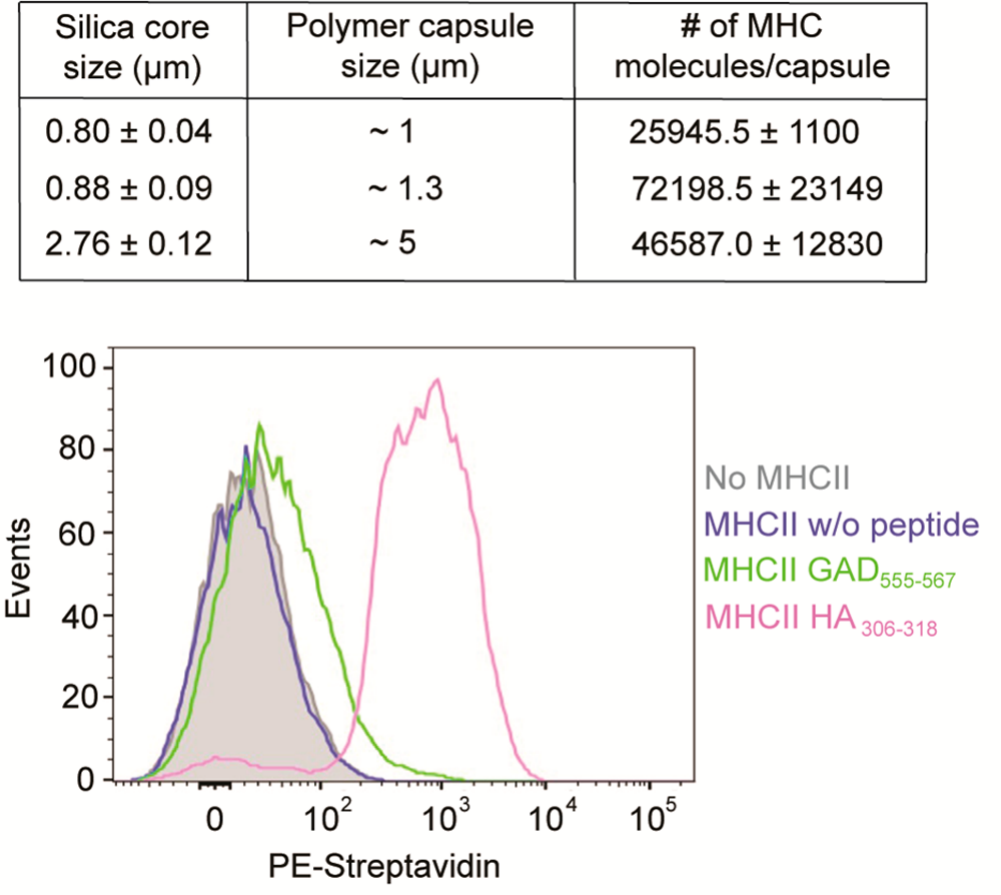
Characterization of pMHC presentation on aAPC. (top) Quantification of MHC II complexes on the capsules (made with 0.8 and 2.76 μm solid-core and 0.88 μm porous core silica). Functionalized 5, 1.3, and 1 μm capsules were prepared, and the amount of MHC II was quantified using quantum simply cellular (QSC) microspheres. The calculated numbers of complexes are indicated. Capsules without MHC II were used as a control. To determine site numbers, the fluorescence intensity of the different capsules was compared with a calibration curve of fluorescence intensity determined for beads carrying known numbers of sites. Data are shown as the mean (±SD) and are representative of at least three independent measurements. (bottom) Flow cytometry analysis of the loaded peptide on the porous silica capsules. Capsules carrying endogenous low-affinity peptides MHC II were loaded with biotinylated peptides. Color code: green, GAD65 peptide; magenta, HA peptide, blue, no peptide; gray, capsules without MHC II. The capsules were labeled with Strep-PE. Only capsules with loaded peptides showed shifts in comparison to capsules with no MHC II or without a peptide.

Next, we quantified the pMHC presentation on the various sizes of LbL-assembled polymer particles that we generated, where we were able to create a range of presentation densities that were roughly 1 order of magnitude lower than the total HLA-DR4 amount on primary APCs^23^ (Figure 2, top, and Figures S5–S7). The polymer particles we developed have the versatility to be directed toward different autoimmune antigens. To that end, we have tested the possibility of loading exogenous peptides on our particles. We prepared polymer capsules presenting MHC refolded with endogenous low-affinity peptides, which were then incubated with either GAD_555-567_ or HA_306-318_ biotinylated peptides specific for the HLA-DR4(*04:01), and the loading was verified using PE-labeled streptavidin (Figure 2, bottom). Flow cytometry (Figure 2) showed that the loading of the HA_306-318_ peptides was much more efficient in comparison to the GAD_555-567_ peptides. These results are consistent with the superior ability of HA-LBL to activate HA-specific clones in comparison to that of GAD-LBL to activate GAD-specific clones. Taking the higher antigen density presented on capsules made with the porous silica core and the potential of loading other molecules into them, we have chosen them for our subsequent analyses.

**Figure 3:**
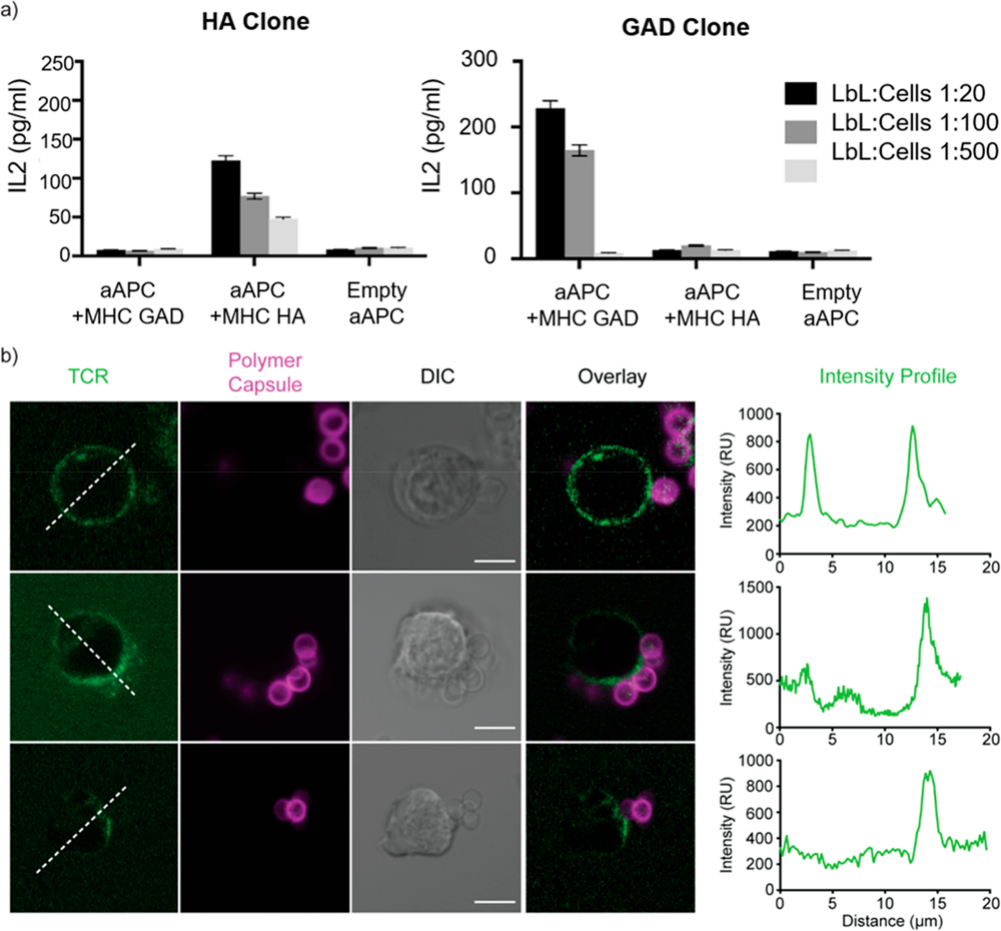
*In vitro* activation of HA or GAD-specific T-cells. (a) Two hybridoma T-cell lines were incubated with aAPC, and IL-2 levels were measured in the supernatants. Error bars are from SEM, three different repeats. (b) T-cell hybridoma polarization. The T-cell receptor (TCR) was labeled with Alexa 488-H57 Fab (green), the cells were mixed with either empty capsules (top) or aAPC (magenta) functionalized with MHC II/GAD complex (middle) or MHC II/HA complex (bottom). TCR polarization was observed in the presence of 5 μm particles carrying the DR4/GAD MHC. Cross-sections along the cell highlight the TCR polarization toward the immunological synapse forming between the cells and the particles. Scale bars: 10 μm. All data were obtained with the 2.76 μm silica core capsules.

**Figure 4:**
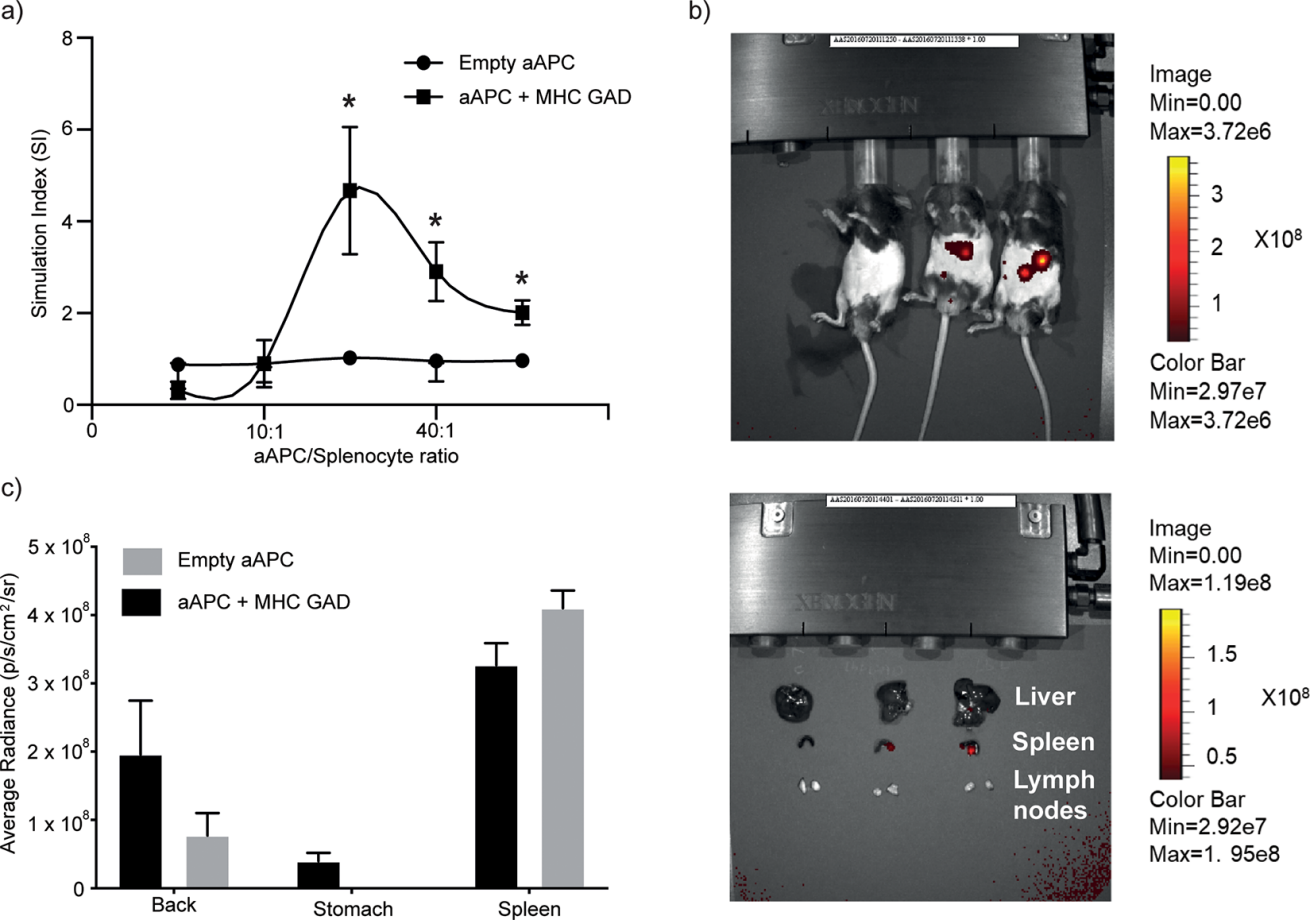
aAPCs target the spleen and result in desensitization of T-cell responses. (a) A recall response assay for splenocytes: the aAPC functionality was performed *ex vivo* on splenocytes harvested from DR4/RipB7 (4.13 T) and from HLA-DR4 transgenic mice as a control. Cells were incubated at different cell to particle ratios as indicated, for 96 h before evaluating proliferation using 3H-thymidine incorporation. Data are shown as mean ± SD (*N* = 6). Multiple unpaired nonparametic *t* test (*, *p* < 0.001279). (b) The *in vivo* biodistribution of the aAPCs was assessed using IVIS imaging. The particles were labeled with maleimide-800. Mice were iv injected with PBS, aAPC presenting MHC/GAD, and empty particles, respectively. (top) Whole-body *in vivo* imaging after 24 h. (bottom) *Ex vivo* images of harvested organs (liver, spleen, and lymph nodes) 24 h postinjection. (c) *Ex vivo* quantification of fluorescence signals of particle accumulation in different organs. All images were obtained by a PerkinElmer IVIS 200 imaging system. Data were obtained with the porous silica core.

The capacity of the aAPC particles to modulate T-cell responses was evaluated *in vitro* using human and mouse models: GAD_555-567_-specific T-cell hybridoma lines G2.1.36.1 and G1.1.7.1 and an HA_306-318_-specific H1.13.2 T-cell hybridoma line (Figure 3). The *ex vivo* functionality was assessed using splenocytes from DR4/RipB7 double-transgenic mice. These mice are transgenic for the costimulatory molecule B7-1 driven by the rat-insulin-promoter and the DR4 allele. They exhibit age-dependent spontaneous loss of tolerance to GAD_555-567_ (which is identical in humans and mice)^24^ (Figure 4). We examined cytokine profiles following the incubation of the T-cell hybridomas with different ratios of polymer particles presenting the cognate pMHC, a nonspecific pMHC, or unloaded (endogenous low-affinity peptides). IL-2 production was only observed with aAPC specifically presenting the cognate pMHC (Figure 3a, Figure S8). To further evaluate the early events leading to T-cell activation, we followed the distribution of the TCR labeled with H57 Fab spm’ upon interaction with the polymer particles. When aAPCs lacking any pMHC are introduced, the TCR is uniformly distributed on the surface of the cells (Figure 3b, top panel). Upon interactions of aAPC with the specific pMHC an immunological synapse is formed, polarizing the TCR toward the particles (Figure 3b, middle and bottom panels).

To evaluate the *in vivo* activity of the engineered GAD_555-567_-specific aAPCs, we examined their ability to induce tolerance using splenocytes from DR4/RipB7 transgenic mice (Figure 4a) and followed their *in vivo* distribution in the different organs (Figure 4b,c). To this end, splenocytes were stimulated by adding a soluble GAD peptide or incubated with aAPC that either carried the GAD_555-567_-loaded MHC or the empty MHC as a control, in an increasing splenocyte/LBL ratio. Splenocyte proliferation was measured 96 h postincubation using labeled thymidine incorporation. Using different ratios of particles to cells, we were able to reach a regime where we could inhibit the proliferation of the cells, as observed in their reduced proliferation index (Figure 4a; average of six mice and S9A for each individual mouse). No responses were observed toward empty particles. Using fluorescently labeled particles that were intravenously injected (iv), we followed the particles’ distribution 24 h postinjection using an *in vivo* imaging system (IVIS). Particles mainly accumulated in the spleen (Figure 4bc and Figure S9b), with no detectable accumulation in the liver or the lymph nodes; hence, we can conclude that they have the potential to reach the target organ in order to induce the tolerance observed *ex vivo*.

**Figure 5:**
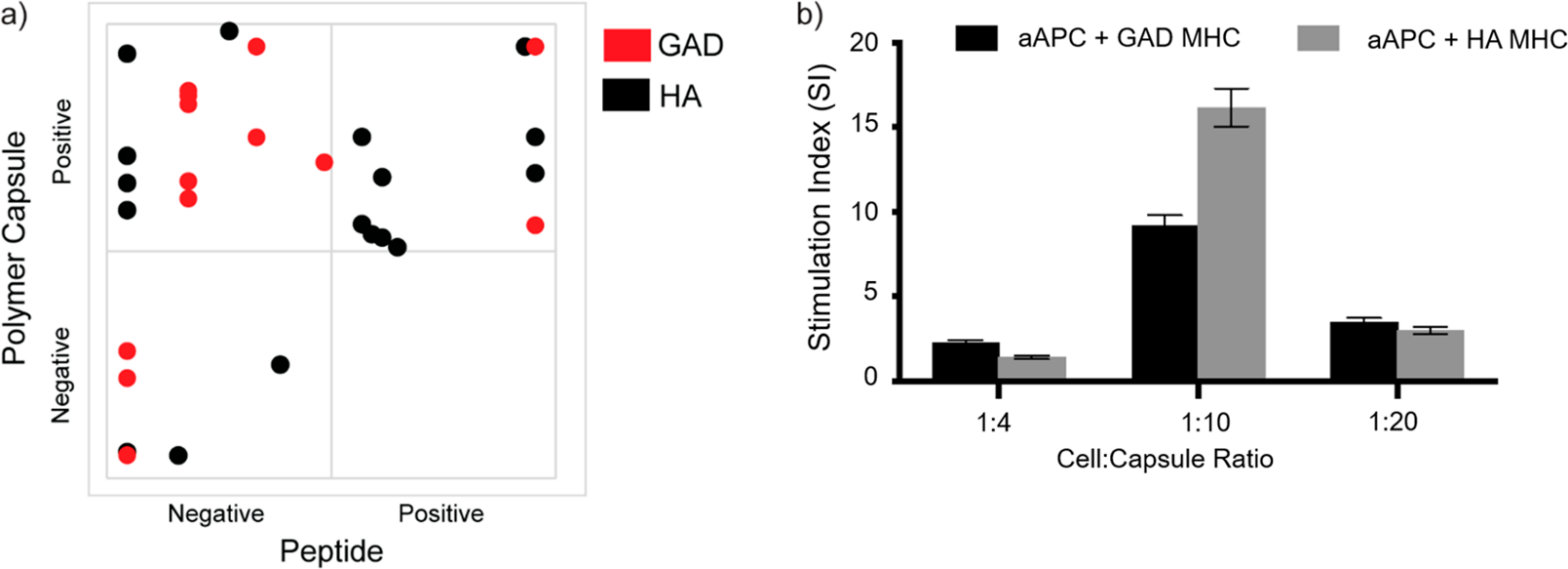
T1D patients’ response to aAPC**.** (a) IFNg positive clones as described using an ELISpot stimulation index for each patient were correlated between samples stimulated with pMHC-aAPC and the same samples stimulated using soluble peptides. Positive responders were defined as those which had a 3-fold increase in response in comparison to the background (either solvent carrier or empty LbL). (b) Stimulation index of two representative patients following stimulation with different aAPC to cell ratios (the exact particle to cell ratio is given in Figure S10). Error bars are from SEM, three different repeats. Data were obtained with the porous silica core.

Finally, we tested the applicability of our engineered particles not only for inducing tolerance but also for enhancing the detection of autoreactive T-cells from human samples. To that end, we used PBMCs derived from HLA-DR4^+^ from recently diagnosed T1D patients and evaluated the production of IFNg using ELISpot (Figure 5a, Figure S10). To evaluate the capacity of our engineered aAPC, we normalized the antigen-specific aAPC response relative to an empty aAPC and compared it to gold-standard assays using peptide-loaded PBMCs. It is clear that the aAPCs were able to detect >50% of patients with GAD_555-567_- or HA_306-318_-responsive cells that were not detected using peptide loading (Figure 5a, positive LbL-negative peptide square), and there was no case where the peptide outperformed the particles. We were also able to observe tolerance induction in the patient samples by varying the ratio between the particles and the cells (Figure 5b); however, unlike the case with mouse samples in which the variability was minimal (Figure 4a), with human samples the exact ratio that induces this phenomenon is patient-specific.

## Discussion

The need for antigen-specific immunotherapy approaches for autoimmune inflammatory diseases is well established. Strategies such as the injection of high doses of soluble peptides, NPs for the manipulation of CD8+ T-cells, and soluble MHC tetramers^25^ have already been shown to be effective in animal models, yet translation into viable treatments in humans has had only limited success. Some of the major drawbacks of the existing techniques are their limited avidity and restricted specificity. Here we report the development of a novel immunotherapeutic tool for detection of and tolerance induction in autoreactive T-cells, using T1D as a model system. We have designed biocompatible and versatile artificial APCs using a layer-by-layer technique, which allows coupling of various proteins to their surface as well as the potential to encapsulate bioactive materials such as drugs and immunomodulatory cytokines. Using our aAPCs, we were able to manipulate responses of T-cells using cell lines as well as primary cells from mouse and patient samples. Furthermore, we have developed a library of aAPC polymer capsules with varying properties, including a variety of surface areas, overcoming a key challenge in previous therapies, which have offered minimal control over the dose and avidity of the stimulus. Our library also has the potential to couple multiple surface molecules beyond the pMHC, such as costimulatory molecules which can affect the immune response. In this work we have used pMHC-coupled polymer capsules presenting an autoantigen associated with type 1 diabetes as well as a viral antigen from influenza as a proof of concept. However, the versatility offered by various possible chemical modifications to the polymers comprising the LbL-assembled polymer particles make them an attractive tool to combine different stimulations such as antibodies, multiple pMHCs, cytokines, chemokines, and costimulatory or coinhibitory molecules to fine-tune the T-cell responses, taking into account the stage of the disease as well as the patients’ needs. The use of high-density aAPC made with the porous silica core allowed us to detect rare populations of autoreactive cells that could not be detected using peptide-pulsed PBMCs, also overcoming any dependence on the specific loading efficiency of peptides of various sequences. Our work is unique in combining the ability to modulate and detect autoreactive T-cells using a relatively simple yet resourceful system.

## Supporting Information Available

Methods detailing the formation of hollow polymer particles, cloning and expressing MHC class II/peptide complexes, cell lines and media, mice, flow cytometry, IL-2 ELISA, a proliferation assay, human samples, cytokine ELISpot, microscopy and figures showing layer-by-layer-assembled polymer particles, the ζ potential of LbL particles, the generation of engineered HLA-DR4/peptide constructs, SIM images of unloaded particles, TEM images of particles of different core sizes, number of HLA-DR molecules on the surface of mature monocyte derived DCs from two different donors, flow cytometry histograms showing the loading of soluble peptides on LbL particles containing “unloaded” MHC, IL2 production using soluble peptides, and antigen-presenting cells, and a recall response assay for splenocytes, and a table summarizing the responses from multiple repeats using human patient samples (PDF)
