# Supplementary material for: Artificial Antigen Presenting Cells for Detection and Desensitization of Autoreactive T cells Associated with Type 1 Diabetes
Source: Nano Lett. 2022 May 26;22(11):4376–82. doi: 10.1021/acs.nanolett.2c00819 (PMC9185737; doi:10.1021/acs.nanolett.2c00819)
Supplement: Supplementary file 1 — nl2c00819_si_001.pdf [file nl2c00819_si_001.pdf]

Artificial Antigen Presenting Cells for Detection and Desensitisation of Auto-reactive T cells Associated with Type 1 Diabetes

Arbel Artzy-Schnirman[*§^1], **Enas Abu-Shah**[*2,3], Rona Chandrawati[1], Efrat Altman[4], Norkhairin Yusuf[5], Shih-Ting Wang[1], Jose Ramos[1], Catherine S. Hansel[1], Maya Haus-Cohen[4], Rony Dahan[6], Sefina Arif[5], Michael L. Dustin[2], Mark Peakman[5], Yoram Reiter[4], Molly M. Stevens[§1]

*co-first author

§ corresponding authors: a_artzyschnirman@rmc.gov.il , m.stevens@imperial.ac.uk

1 Department of Materials, Department of Bioengineering and Institute for Biomedical Engineering, Imperial College London, Prince Consort Road, London SW7 2AZ, UK

2 Kennedy Institute of Rheumatology, Nuffield Department of Orthopaedics, Rheumatology and Musculoskeletal Sciences, University of Oxford, Oxford OX3 7FY, UK

3 Sir William Dunn School of Pathology, University of Oxford, Oxford OX1 3RE, UK

4 Laboratory of Molecular Immunology, Faculty of Biology and Technion Integrated Cancer Center, Technion-Israel Institute of Technology, Haifa, 3200003, Israel

5 Department of Immunobiology, Guy's, King's & St Thomas' School of Medicine, 2nd Floor, New Guy's House, Guy's Hospital, London SE1 9RT, UK

6 Department of Immunology, Weizmann Institute of Science, Rehovot, 7610001, Israel

^ Present affiliation: Applied Medical Technology Research Center, Rambam Health Care Campus, Haifa, 3109601 , Israel

Supporting Information

Methods

Formation of hollow polymer particles

Hollow polymer particles assembled by the layer-by-layer technique were prepared as previously described [1]. Briefly, silica particles (50 mg/ml) were first washed with three centrifugation/redispersion cycles (1000 g, 30 s) with 20 mM NaOAc buffer pH 4.0. Assembly of polymer multilayers was achieved by alternately incubating the particles with PVP (1 mg/ml in 20 mM NaOAc buffer pH 4.0, 15 min) and PMA_{SH} (1 mg/ml in 20 mM NaOAc buffer pH 4.0, 15 min). The particles were washed three times (1000 g, 30 s) with NaOAc buffer between layers and the process was repeated until five bilayers of PMA_{SH}/PVP were assembled. The thiols within the polymer layers were crosslinked with 1,8-bis(maleimido)diethylene glycol ((BM)PEG_2, 1 mM in 50 mM MES buffer pH 6.0, overnight). Hollow polymer shells were obtained by dissolving the silica cores using a 2 M hydrofluoric acid (HF)/8 M ammonium fluoride (NH_4F) solution for 2 min, followed by multiple centrifugation/washing cycles (4500 g, 3 min). *Caution! HF and NH4F are highly toxic. Handling HF and NH4F solutions should be done with extreme caution, and only small quantities should be prepared*Preparation of Alexa Fluor 488-labelled PMA_{SH}: A solution of PMA_{SH} (10 mg mL^{-1}) was incubated with 100 µL of Alexa Fluor 488 maleimide (1 mg mL^{-1}). The reaction was allowed to proceed overnight and excess Alexa Fluor 488 was removed through purification by centrifugal filter devices.

LbL concentration of each batch was determined using Flow Cytometry Cell Counting Beads (Thermo fisher, C36950).

PMA_{SH} was synthesized according to previously published protocols[1]. In brief, a PMA solution (250 mg of 30 wt % solution) was diluted into 5 mL of potassium phosphate buffer (0.1 M, pH 7.2), charged with EDC (70 mg) and NHS (45 mg) and was stirred for 15 min. This was followed by the addition of 7.5 mg of cysteamine hydrochloride, which had been preoxidized in air for several days. The reaction was allowed to proceed overnight. After dialyzing against distilled water, the polymer was isolated by freeze-drying. By reprecipitating from water into dioxane, the final solid was purified further. Elemental analysis of the polymer was used to estimate the degree of functionalization.

Cloning and expressing MHC class II/peptide complexes

Plasmids for the expression of recombinant four-domain MHC class II DR4 molecules in Schneider S2 cells, a gift from Dr. Lars Fugger, DR-A1*0101/DR-B1*0401, DR-A1*0101/DR-B1*0401(HA-306-318) and DR-B1*0401(GAD-555-567), have been previously described [2]. Site-directed mutagenesis (Quick change II, Agilent 200523) was used to introduce the C-terminus cysteine mutation in the DR-A chain using the following primers: DRA5'cys(GAAGATCGAGTGGCACTGTTAAAAGGGCAATTCTG), DRA3'cys(CTTCTAGCTCACCGTGACAATTTTCCCGTTAAGAC). DR-A and DR-B plasmids were co-transfected with pCoBlast selection vector to S2 cells using cellfectin reagent (invitrogen). Stable single-cell line clones were verified for protein expression. Upon induction with CuSO4, cell supernatants were collected and DR4 complexes were affinity purified by anti-DR LB3.1 (ATCC number HB-298) mAb. The purified DR4 complexes were characterized by SDS-PAGE. The correct folding of the complexes was verified by recognition of anti-DR conformation sensitive mAb (L243) in an ELISA binding assay[3].

Peptides for loading and functional assays were synthesized by standard fluorenylmethoxycarbonyl chemistry and purified to >95% by reverse phase HPLC, $GAD_{555-567}$ (NFFRMVISNPAAT) $HA_{306-318}$ (PKYVKQNTLKLAT) or bought from Gene Script.

Cell lines and media

The Preiss cell line (EBV-transformed B-lymphoblast), H2-1 T-cell hybridomas, DR*1501 L cells transfectant (L466.1) and CTLL cell lines were maintained in complete media in RPMI-1640 supplemented with 10 v/v fetal calf serum (FCS), 2 mM glutamine, Penicillin (100 units/ml) and Streptomycin (100 µg/ml).

The G2.1.36.1 and H1.13.2 T-cell hybridomas were maintained in Dulbecco's modified Eagles medium (DMEM) and with 10 v/v FCS, 2mM glutamine and 1v/v Penicillin Streptomycin antibiotics.

Mice

All experiments were performed in accordance with the Israeli laws and approved by the ethics committee at the Technion-Israel Institute of Technology. DR0401-IE mice12- Class II deficient

C57Bl/6 mice (I-Abo/o) transgenic for the DRA1*0101 and DRB1*0401 genes. These mice express a human-mouse chimeric class II molecule in which the TCR interacting and peptide binding domains of mouse I-E (domains α1 and β1, exon 2 in both genes) have been replaced with the α1 and β1 domains from DRA1*0101 and DRB1*0401, respectively. Retention of the murine α2 and β2 domains allows for the cognate murine CD4-murine MHC interaction.

RIP-B7/DR0401 - C57Bl/6 mice transgenic for the costimulatory molecule B7-1 driven by the rat-insulin-promoter (RIP-B7 mice) were crossed with DR0401-IE mice to generate RIP-B7/DR0401-IE (B7/DR0401) mice. These mice are spontaneously diabetic with disease incidence of ~30% at 54 weeks and a mean diabetes onset age of 37 ± 9 weeks. These mice exhibit age-dependent spontaneous loss of tolerance to 2 islet Ags $GAD_{555\text{-}567}$ (identical sequence in human and mice) and GFAP-240-252 during the pre-diabetic and diabetic phases.

All mice in the *ex vivo* and *in vivo* experiments were 8 weeks when the experiments were performed.

Flow cytometry

Quantification of pMHC on antigen presenting cells (B-cell line, or monocyte derived dendritic cells) or on LbL particles was performed using the Quantum™ Simply Cellular® (QSC) microspheres and an anti HLA-DR4 PE-conjugated mAb (L243). Samples were analyzed on a X20 Fortessa or LSRII flow cytometer (BD Biosciences).

IL-2 ELISA

T-cell hybridoma cells ($2x10^5$/well in a 96-well plate) in 100 µl of 10% FBS-containing medium were combined with pMHC LbL at different ratios or as a control with $2x10^5$ DR4-EBV-transformed DR4 positive B lymphoblast MGAR cell line in 100 µl alone or in the presence of peptides in the indicated concentration. Cells were incubated at 37 °C and 5% CO_2. After 24 h of culture. The presence of supernatants IL-2 was measured by sandwich Elisa. ELISA plates were coated with anti-IL-2 Ab (BioLegend) at a concentration of 0.25ug/ml overnight at 4 °C or 1 h at 37 °C. The plates were blocked for 30 min at room temperature with 5 v/v FCS-PBS and subsequently were incubated with the hybridoma supernatants for 2 h at room temperature. Then a biotinylated anti-IL-2 Ab (BioLegend) at a concentration of 0.25 mg/ml was added and after washing, plates were incubated with Strp-HRP antibody.

Proliferation assay

Splenocytes from immunized mice were isolated and then cultured in a density of 5*10^5 cell/well in triplicates with or without peptide antigens for 4 days in a stimulation media (DMEM supplemented with 10 v/v FBS, 2 mM sodium pyruvate, 2 mM l-glutamate, 4 mM 2-mercaptoethanol, and 50 µg/ml penicillin/streptomycin) with GAD-LBL or empty-LBL at splenocytes to LBL ratio as indicated. Proliferation responses were assessed during the last 16 hours of incubation with 3H-thymidine. On glass fiber filters, cultures were harvested and 3H-thymidine uptake was measured using liquid scintillation. By dividing cpm of peptide-stimulated cultures by cpm of control cultures, the stimulation index (SI) was calculated.

Human samples

PBMCs from 11 diagnosed patients with T1D were collected. All patients were HLA-DR4+. Fresh and frozen samples were compared with no obvious difference. An ethical approval was obtained from each participating center's local research ethics committee (National Research Ethics Committee, Bromley NRES Committee, reference number 08/H0805/14) and all participants provided written informed consent.

Cytokine ELISpot

Fresh or frozen PBMCs were dispensed into 48-well plates at a density of 2 × 106 in 0.5 ml in RPMI-1640 supplemented with antibiotics Pen/Strep (TC medium; Life Technologies Ltd.) and 10 v/v human AB serum (Sigma) supplemented with peptides (GAD, HA, and INFANRIX) or carrier solvent (DMSO or PBS respectively) to a final concentration of 10 µM or the LBL in different ratios, and incubated at 37 °C, 5% CO_2, tilted by 5°. Control wells contained TC medium with an equivalent concentration of peptide/LBL. On day +1, 0.5 ml prewarmed TC medium/10% AB serum was added, and on day +2, nonadherent cells were resuspended using prewarmed TC medium/2% AB serum, washed, brought to a concentration of 106/300 µl, and 100 µl dispensed in triplicate into wells of 96-well ELISA plates (Nunc Maxisorp; Merck Ltd., Poole, United Kingdom) preblocked with 1w/v BSA in PBS and precoated with monoclonal anti–IFN-γ (U-Cytech, Utrecht, The Netherlands). When sufficient cells were available, IFN-γ was analyzed for each test peptide/ LbL. After capture at 37°C, 5% CO_2 for 22 hours, cells were lysed in ice-cold water, plates washed in PBS/Tween 20, and spots developed according to the manufacturer's

instructions. Plates were dried and spots of 80–120 µm counted in a BioReader 3000 (BioSys, Karben, Germany).

ELISpot analysis was done by individual blinded to the stimulation condition. A threshold of 3 clones was set up to account for a meaningful positive response. Samples stimulated with pMHC LbL were normalised to the response to LbL without MHC, and for the peptide stimulation the samples were normalized to solvent. To account for donor variability, the data was normalised to the maximum response of each donor.

Microscopy

3D SIM (structured illumination microscopy) imaging was performed at room temperature using an Elyra PS.1 (Carl Zeiss).The images were acquired using a 63x, 1.4 NA oil-immersion objective lens, with three orientation angles of the excitation grid and five phases per image, with a 110 nm z-step and a 64 nm pixel size, all imaged at 16 bits per pixel with an Andor Zyla. SIM data was processed by the SIM module of the Zen software package (Carl Zeiss), then exported as TIF stacks. The SIM data sets were then processed into projection images using ImageJ software.

Transmission electron microscopy (TEM) imaging. All imaging was performed with JEOL 2100F TEM, with an acceleration voltage of 200 kV. For sample preparation, 10 µl of the particle solutions were dropped on the 200 mesh copper grid covered with a carbon-stabilized Formvar film (Electron Microscopy Sciences). The residual solution was removed after 5 min. The sample with LbL polymer shells was negatively stained by 5 µl of 1 wt% uranyl acetate solution on the grid for 5 min.

Scanning confocal microscopy was performed using a 63x silicon oil objective on an Olympus FV1200. T-cells were labelled with H57-Alexa488 Fab for 20 min before a brief wash at 4 ^{o}C to remove unbound Fab, followed by a 10 min incubation with LbL particles before imaging.

Bibliography

(1) Chandrawati, R. Layer-by-Layer Engineered Polymer Capsules for Therapeutic Delivery. In *Methods in Molecular Biology*; 2018; Vol. 1758, pp 73–84. https://doi.org/10.1007/978-1-4939-7741-3_6.

(2) Svendsen, P.; Andersen, C. B.; Willcox, N.; Coyle, A. J.; Holmdahl, R.; Kamradt, T.; Fugger, L. Tracking of Proinflammatory Collagen-Specific T Cells in Early and Late

Collagen-Induced Arthritis in Humanized Mice. *J. Immunol.* **2004**, *173* (11), 7037–7045. https://doi.org/10.4049/jimmunol.173.11.7037.

(3) Finn, T. P.; Jones, R. E.; Rich, C.; Dahan, R.; Link, J.; David, C. S.; Chou, Y. K.; Offner, H.; Vandenbark, A. A. HLA-DRB1*1501 Risk Association in Multiple Sclerosis May Not Be Related to Presentation of Myelin Epitopes. *J. Neurosci. Res.* **2004**, *78* (1), 100–114. https://doi.org/10.1002/jnr.20227.

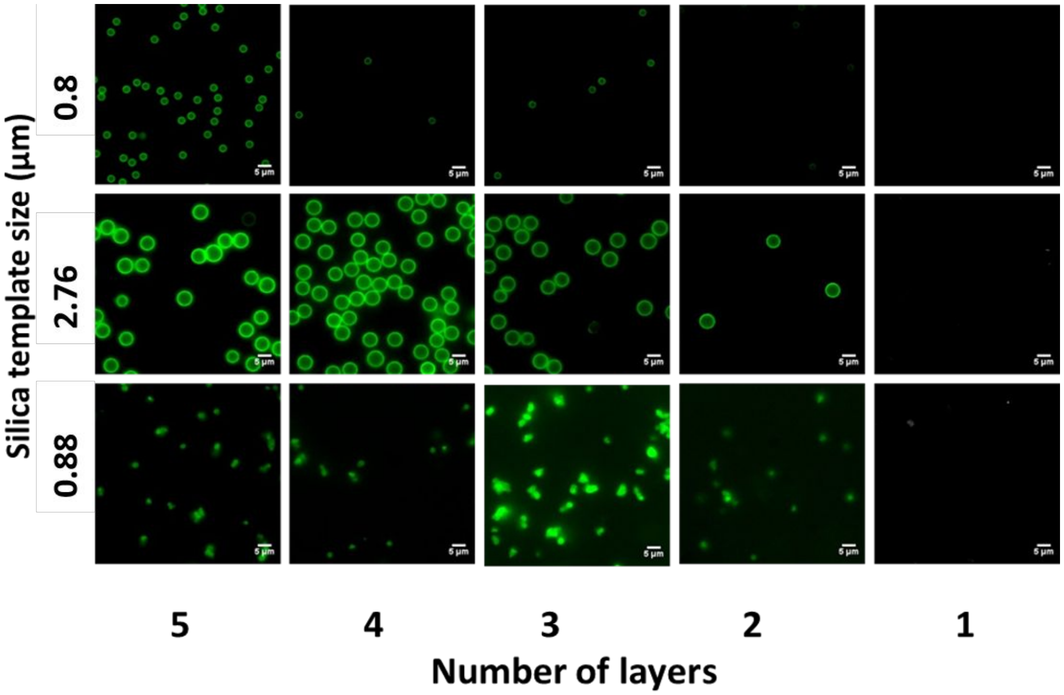

Figure S1: Layer-by-layer assembled polymer particles. PVP and fluorescently labelled PMA_{SH} were sequentially deposited on silica particle templates and confocal images were taken after each deposition step. There is an obvious increase in fluorescence as the layering rounds progress. Note the decrease after the 3rd round for the porous silica core (0.8 µm) which is probably a result of self-quenching of the fluorophore due to its high surface density.

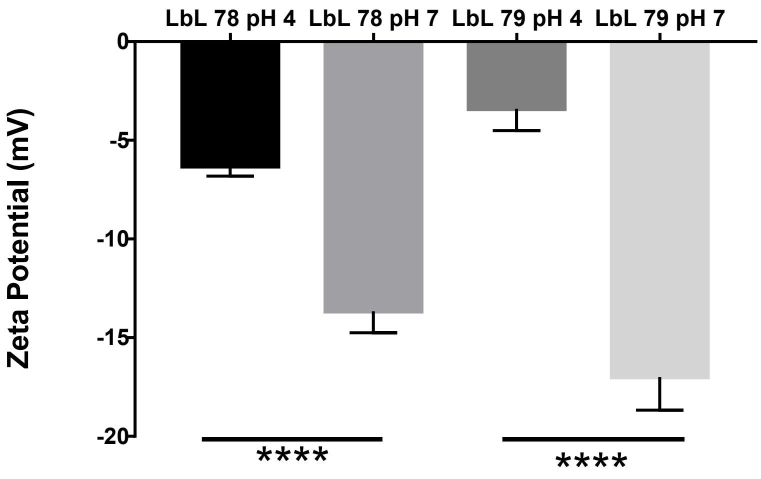

Figure S2: Zeta potential of LbL particles. The zeta potential was measured for two batches of LbL particles (labelled 78 and 79) at pH 4 where the particles were still compact and at pH 7 after the particles' core expanded. Data shown as mean ± S.D., n = 3 , Statistical test, **** $p < 0.0001$.

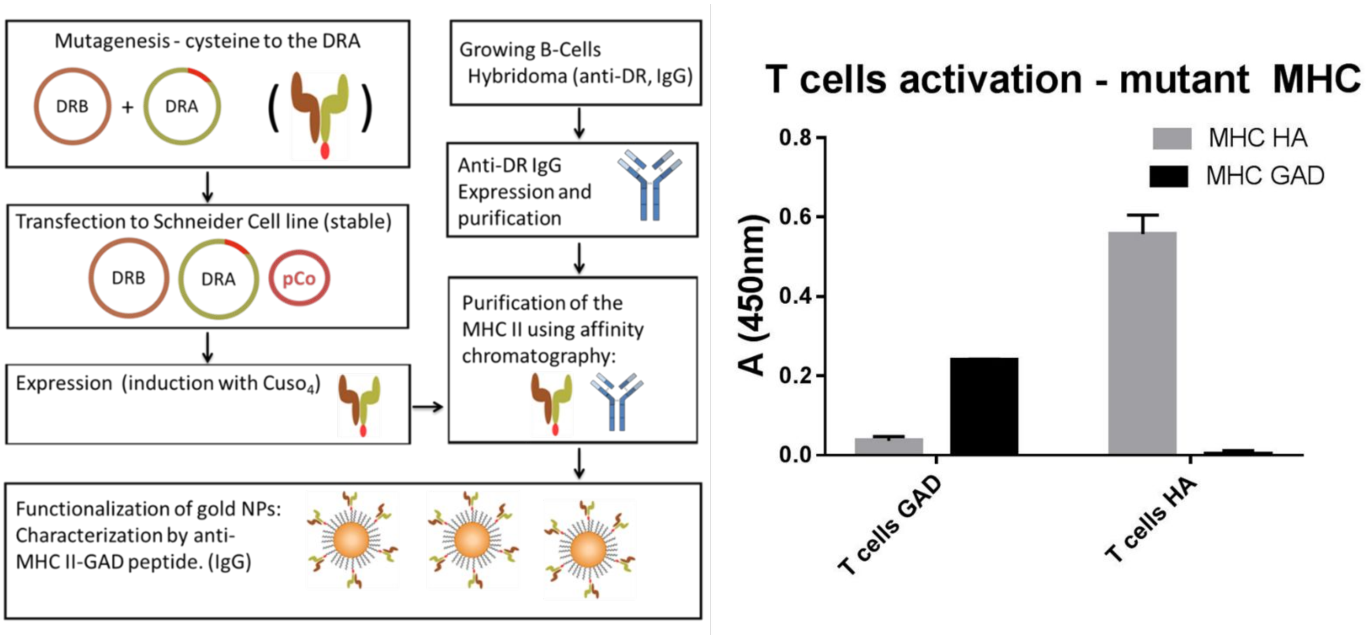

Figure S3: Generation of engineered HLA-DR4/peptide constructs. A) Schematic representation of the generation of cysteine modified HLA-DR4, expression in insect cells and purification of correctly refolded monomers using a confirmation sensitive antibody. The fully functional pMHC is then coupled to our particles through disulfide bonds. B) Validation that the T-cell respond well to the (cysteine) mutated MHC. Data shown as mean ± S.D., n = 3.

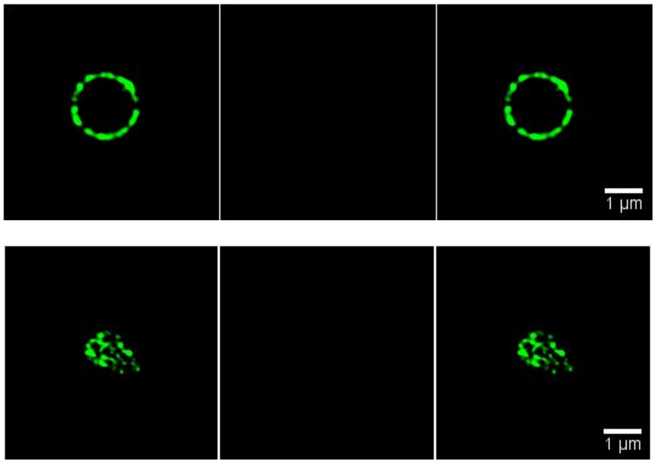

Figure S4: SIM images of unloaded particles. Top panel shows the fluorescent LbL on 2.7 µm core particles, bottom panel is with the porous silica. The middle panel shows the lack of MHC signal after labelling with the anti-DR4 antibody.

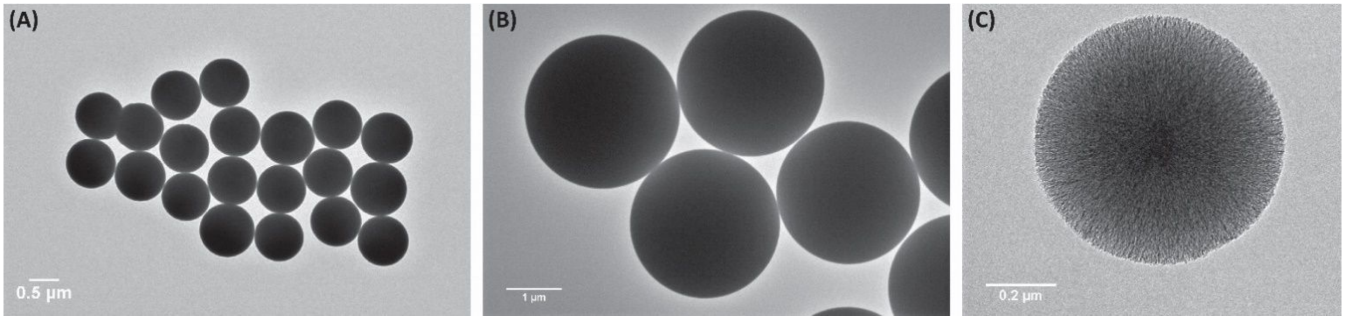

Figure S5: TEM images of particles of different cores size: (A) 800 nm, (B) 2700 nm and (C) porous silica.

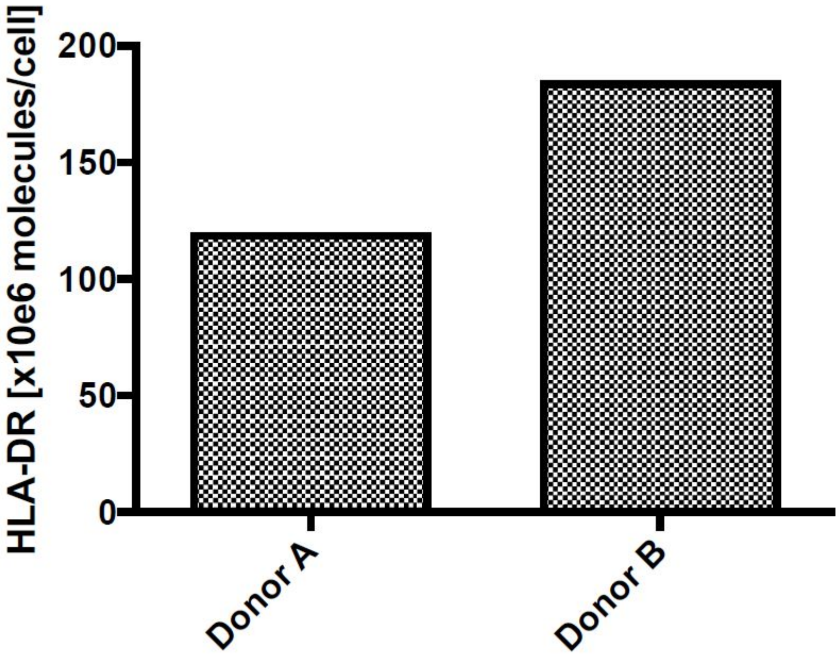

Figure S6: Number of HLA-DR molecules on the surface of mature monocyte derived DCs from two different donors.

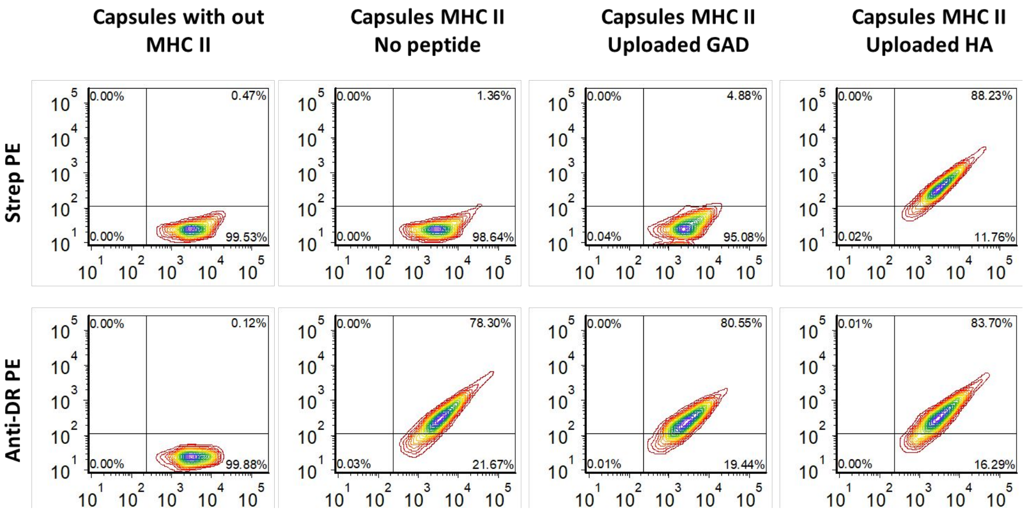

Figure S7: Flow cytometry histograms showing the loading of soluble peptide on LbL particles containing "unloaded" MHC. The x axis is the signal from the labelled PMA_{SH} layer (Alexa Fluor 647), x axis in the top panel is streptavidin PE (binds to biotinylated peptides) and bottom panel is an anti HLA-DR4 PE specific antibody. Note the correlation between the number of layers (APC intensity) and the amount of MHC (PE intensity lower panel), also note the low loading efficiency of the GAD peptide.

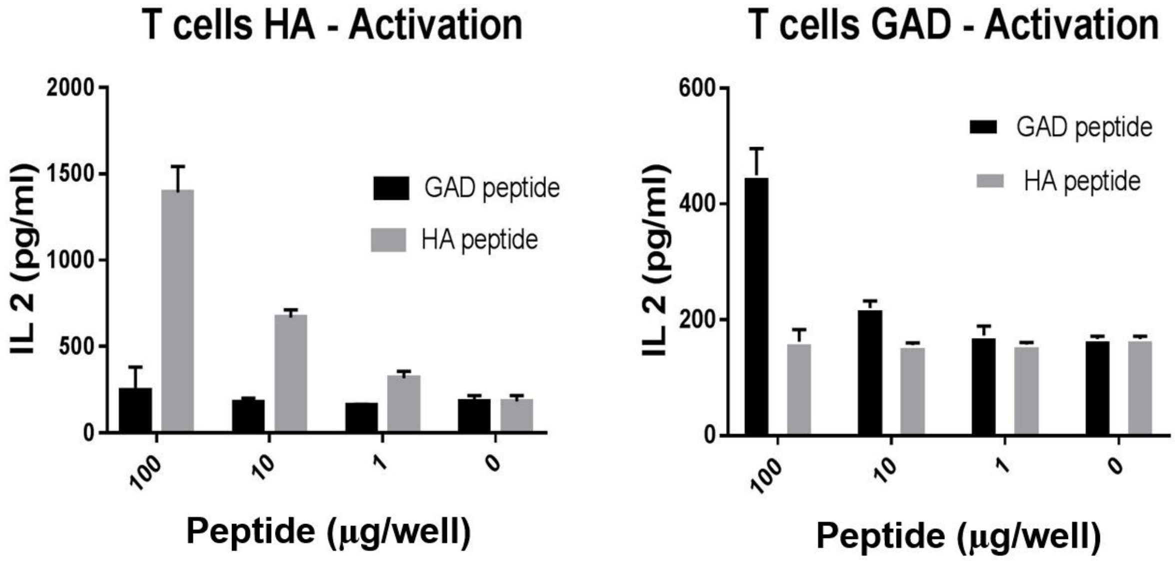

Figure S8: IL2 production using soluble peptides and antigen presenting cells demonstrating the lower activation of the GAD cell lines (lower IL2 production for the same amount of peptide as the HA) Data shown as mean ± S.D., n = 3.

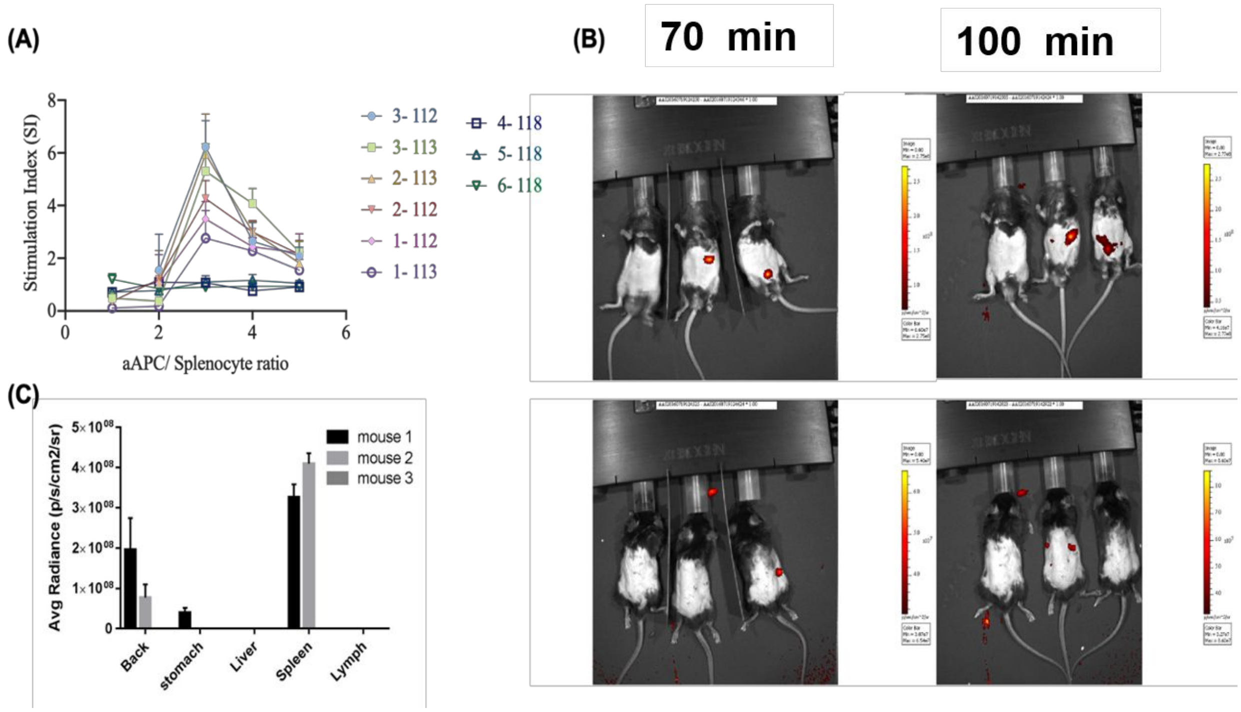

Figure S9: (A) A recall response assay for splenocytes the LbL particle functionality was performed ex vivo on splenocytes harvested from DR4/RipB7 (4.13 T) and from HLA-DR4 transgenic mice as a control. Cells were incubated at different cell: particle ratios as indicated, for 96 h before evaluating proliferation using 3H-thymidine incorporation. Experiments are average of 6 independent mice. (B) IVIS imaging of particle distribution 70 and 100 min after injection. The bright spot where a significant number of particles accumulates. (C) Quantification of particle accumulation in different organs (mouse 1: GAD particle; mouse 2: empty particles; mouse 3: PBS control).

	Patient number	controls				SI	
		HA	GAD	INFANRIX	Cells:LbL	LbL HA	LbL GAD
1 Background no MHC	MP033 30.6.2015	-	-	+++	1:1	-	-
					4.7:1		
					47:1		
2	2016	+	-	+++	2.55:1	+	+
					10:1	-	-
3	4007	missing		+++	2.55:1	-	-
					10:1	+++	+++
**4**	2027 (Fresh)	-	-	+++	4.3:1	-	-
					8.7:1	+++	++
					17.4:1	-	+
	2027	-	-	+++	1	+	-
					2	++	-
					3	-	-
**5**	2011	-	-	+++	4.3:1	-	-
					8.7:1	-	-
					17.4:1	+	+++
6	2001	+	-	+++	4.3:1	-	++
					8.7:1	+++	++
					17.4:1	+++	Missing
7	2010	-	-	+++	4.3:1	-	++
					8.7:1	-	++
					17.4:1	+	
	2010 repeat	+	-	+++	1	+	-
					2	-	-
					3	-	-
8	2018	+	-	+++	4.3:1	-	-
					8.7:1	-	+++
					17.4:1	++	+
9	2021	+	-	+++	1	+++	+
					2	+++	+
					3	-	-
10	2025	missing	+	+++	1	missing	-
					2		++
11	4001	+++	-	+++	1	+++	+
					2	+++	++
					3	+++	++
12	2031	-	-	+++	1	-	-
					2	+++	+++
					3	+	+

Figure S10: A summary table of the responses from multiple repeats using human patient samples. The first sample (patient MP033) is the response detected when the donor is known to be DR4 negative, so no response is recorded. The control columns refer to the addition of soluble peptides or INFRANIX as a positive control for activation. No response (-) is determined as a response which is less than three folds above the no peptide background (or no MHC for the LbL samples). (+, ++ and +++ refer to responses of varying strength from low to very high). The cell to LbL ratios differed between different donors and different batches and hence the information there should be taken for qualitative purposes only.
